# High Expression of circ_0001821 Promoted Colorectal Cancer Progression Through miR-600/ISOC1 Axis

**DOI:** 10.1007/s10528-022-10262-z

**Published:** 2022-08-09

**Authors:** Cheng Li, Xudong Gao, Yi Zhao, Xin Chen

**Affiliations:** 1 Department of Surgical Oncology, Shaanxi Provincial People’s Hospital, Xi’an, Shaanxi China; 2 Department of Otolaryngology, Shaanxi Provincial People’s Hospital, Xi’an, Shaanxi China; 3 Department of Radiotherapy, Shaanxi Provincial People’s Hospital, No. 256 Youyi West Rd, Xi’an, 710068 Shaanxi China

**Keywords:** Colorectal cancer, circ_0001821, miR-600, ISOC1

## Abstract

It has been reported that circRNAs play an important regulatory role in the progression of colorectal cancer (CRC). However, the molecular role of circ_0001821 in CRC development is unclear. In this study, we aimed to investigate the regulatory role and molecular mechanisms of circ_0001821 in CRC. Reverse transcription-quantitative PCR and western blot assays were used to detect the expression of circ_0001821, miR-600 and isochorismatase domain containing 1 (ISOC1) in CRC tissues as well as its cell lines. Colony formation assay and EDU assay were used to detect the proliferative capacity of cells. Transwell assay was used to assess cell migration and invasion ability. Flow cytometry was used to analyze cell apoptosis. ELISA was used to measure the glycolytic capacity of cells. Dual-luciferase reporter assay and RNA pull-down assay were used to analyze the relationships between circ_0001821, miR-600 and ISOC1. Animal experimentation was used to validate the functional study of circ_0001821 in vivo. Immunohistochemistry (IHC) of Ki67 staining analysis was conducted to assess tumor growth. Circ_0001821 and ISOC1 were significantly increased in CRC tissues and its cell lines, and miR-600 was significantly decreased in CRC tissues and its cell lines. Silencing circ_0001821 inhibited cell proliferation, migration, invasion and glycolytic capacity, while inducing apoptosis. And it could inhibit tumor growth in vivo. Circ_0001821 could act as a sponge for miR-600 to regulate CRC processes. ISOC1 was identified as a downstream regulator of miR-600, also miR-600 could regulate the expression of ISOC1. In addition, circ_0001821 could regulate ISOC1 expression changes through miR-600. Mechanistically, either miR-600 inhibitor or overexpression of ISOC1 could reverse the effects of knockdown of circ_0001821 on cell biological properties. Circ_0001821 regulated the developmental process of CRC through miR-600/ISOC1 axis.

## Introduction

Colorectal cancer (CRC) is a common malignant disease and an important problem that has seriously endangered the health of the population worldwide (Bonney et al. 2021; Henderson et al. 2021; Kordahi et al. 2021). In addition, CRC is the second most important contributor to death due to cancer (Ferlay et al. 2015; Sun et al. 2019). The disease is also affected by lifestyle habits, diet, living environment, and genetic factors (Jung et al. 2021; Stefani et al. 2021). The number of male patients with CRC is much higher than that of female patients (Njor et al. 2021; Wekha et al. 2021). The age of CRC incidence is increasing year by year, and the incidence is becoming more intensive in middle and old age (Alyabsi et al. 2021; Lazarova and Bordonaro 2021). CRC has a good prognosis if it can be detected earlier and treated surgically (Li et al. 2021). If the disease is already at an advanced stage, it can be treated with surgery, but the postoperative complications are also very frightening and directly related to the mortality of CRC (Bonney et al. 2021; Henderson et al. 2021; Kok et al. 2021; Zhong et al. 2021). However, the molecular mechanism of CRC in the pathogenesis was not clear. Therefore, our study aimed to discover biomolecular markers of CRC that could be better, earlier and more precise.

Circular RNAs (circRNAs) are a large class of novel non-coding RNAs. CircRNAs are stably expressed in cells and have stable closed-loop structures that are not easily degraded (Chen et al. 2021a). They attract much attention in the current research field because of their structural peculiarities. Some studies have reported that circRNAs are dysregulated in many cancers and have important regulatory roles (Bi et al. 2019; Wang et al. 2018). Besides, DNA methylation plays an important role in various biological processes in prokaryotes and eukaryotes, and circRNAs are formed by reverse splicing of the corresponding host genes and can therefore be regulated by methylation (Chen and Yang 2015; Xing et al. 2014). On the other hand, microRNAs (miRNAs) are also a hot topic of current research, as they are easily detected in cells or body fluids and also have important regulatory roles (Fonseca et al. 2021; Raut et al. 2021). There are many reports that circRNAs can act as sponges of miRNAs and co-regulate the progression of cancer. For example, circ-ZKSCAN1, which was significantly dysregulated and reduced in expression in bladder cancer, was a potent molecular bio-marker, in addition, circ-ZKSCAN1 could further regulate the progression of bladder cancer by acting as a sponge for miR-1178 (Bi et al. 2019); circ_0008305 had decreased expression in lung cancer and could also act as a sponge for miR-429 and miR-200b to further regulate lung cancer progression (Wang et al. 2018). Song et al. showed that circ_0001821 expression was significantly upregulated in CRC (Song et al. 2021), but the specific function of circ_0001821 in the development of CRC is unclear. In the study by Zhang et al. showed that miR-600 expression was dysregulated and significantly reduced in CRC (Zhang et al. 2017). However, the regulatory network of circ_0001821/miR-600 axis in CRC was not clear, and we aimed to investigate the regulatory role of circ_0001821/miR-600 axis in CRC.

Circ_0001821 first appeared in the study of Wang et al. and its expression was significantly dysregulated in human lung adenocarcinoma and squamous cell carcinoma (Wang et al. 2019). However, whether circ_0001821 is dysregulated in CRC and the potential biomolecular pathogenesis of circ_0001821 in CRC progression were unknown. No functional studies of circ_0001821 in CRC were available at this stage. In our present study, we first examined the expression of circ_0001821 in CRC and further investigated the specific functions of circ_0001821 in the development of CRC. Our results suggested that circ_0001821 might be a new therapeutic target in the diagnosis and treatment of CRC.

## Materials and Methods

### Tissue Specimens

All CRC tissue samples as well as normal tissue samples for this study were obtained from Shannxi Provincial People's Hospital. All patients signed a written informed consent before the procedure was performed. In addition, all tissues were placed in liquid nitrogen immediately after removal and then frozen in a − 80 °C refrigerator. The study was approved by the Shannxi Provincial People's Hospital Ethics Committee.

### Cell Culture

Human normal colonic epithelial cells (NCM460; #BNCC339288) was purchased from BeNa Culture Collection (Beijing, China) and CRC cell lines (SW620 and HCT116; #CL-0225B and #CL-0096) were purchased from procell (Wuhan, China). The medium used for all cells was RPMI 1640 (Invitrogen, Carlsbad, CA, USA) and 10% fetal bovine serum (FBS; Invitrogen) was added to the medium. In addition, an incubator containing 5% CO_2_ and 37 °C was used as the culture condition for all cell lines.

### Cell Transfections

Small interference RNA against circ_0001821 (si-circ_0001821) and matched negative control (si-NC) were provided by Geneseed (Guangzhou, China). MiR-600 mimic (miR-600; #miR10003268-1-5), mimic NC (miR-NC; #miR1N0000001-1-5), miR-600 inhibitor (anti-miR-600; #miR20003268-1-5) and inhibitor NC (anti-miR-NC; #miR2N0000001-1-5) were provided by Ribobio (Guangzhou, China). Isochorismatase domain containing 1 (ISOC1) overexpression vector (ISOC1) and blank vector control (pcDNA) were also constructed by Ribobio. Cells were transfected with vectors or oligonucleotides using Lipofectamine 3000 reagent (#L3000015; Invitrogen).

### Reverse Transcription-Quantitative PCR (RT-qPCR)

TRizol (#15596018; Invitrogen) was used for lysis of total RNA in all tissues as well as in all cell lines. The concentration of extracted total RNA was then measured and recorded using a UV spectrophotometer (Xipu, Shanghai, China). The extracted total RNA was further reversely transcribed to cDNA using a 1st Strand cDNA Synthesis Kit (#11119ES60; Yeasen, Shanghai, China) or miRNA 1st Strand cDNA Synthesis Kit (#11148ES10; Yeasen). Finally, PCR reactions were performed using SYBR Green mix (#11202ES03; Yeasen). Among them, U6 and β-actin were used as internal references, normalized and calculated by the 2^−ΔΔCt^ method. Primer sequences are listed in Table 1.

**Table 1:** Primers sequences used for PCR

Name		Primers for PCR (5ʹ–3ʹ)
hsa_circ_0001821	Forward	GGGTCTCCCTATGGAATGTAAGAC
	Reverse	GCCAAAAGATCAGGCCTCAAGC
miR-600	Forward	GCCGAGACTTACAGACAAGAG
	Reverse	CTCAACTGGTGTCGTGGAG
ISOC1	Forward	TCGACATGCACCGCAAATTC
	Reverse	AGTGAGCTGGATCTGCAACG
β-actin	Forward	CTTCGCGGGCGACGAT
	Reverse	CCACATAGGAATCCTTCTGACC
U6	Forward	CTCGCTTCGGCAGCACA
	Reverse	AACGCTTCACGAATTTGCGT

### Colony Formation Assay

SW620 and HCT116 cells transfected with plasmids were inoculated in 96-well plates (5 × 10^3^ cells/well). After the cells were grown stably against the wall, the medium was replaced every two days. After culturing cells for 12 days, the medium was discarded and washed with PBS. The cell colonies were then stained with crystal violet (#A100528-0025; Sangon, Shanghai, China), and then manually counted and recorded after imaging using an inverted microscope.

### EDU Assay

The proliferation ability of the cells was tested using the EDU cell proliferation assay kit (#C10310-1; Ribobio). Firstly, SW620 and HCT116 cells transfected with plasmids were inoculated in 24-well plates (8 × 10^4^ cells/well), and after the cells were grown stably, EDU reagent was added to the cells and incubated for a total of 3 h with gentle shaking. The nuclei were stained using DAPI reagent (#DA0001; Leagene, Shanghai, China). Finally, the cells were placed under an inverted fluorescence microscope for imaging processing. The number of EDU positive cells on the pictures was then counted and recorded for analysis.

### Transwell Assay

The migration and invasion abilities of the cells were assayed by transwell assay, and the chambers coated without or with Matrigel (Corning Incorporated, Corning, NY, USA) were used to evaluate cell migration or invasion, respectively. SW620 and HCT116 cells transfected with plasmids in serum-free medium were inoculated into the upper chamber. Then medium with 10% FBS was added in the lower chamber. Finally, the upper and lower chambers were placed together for co-culture. After waiting for 24 h, the cells that migrated or invaded into the lower chamber were stained with crystal violet, then images were acquired using an inverted fluorescence microscope, and finally the pictures were counted and recorded.

### Flow Cytometry

Apoptosis was detected using the Annexin V-FITC/PI Apoptosis Detection Kit (#CA1020; Solarbio, Beijing, China). Plasmid-transfected SW620 and HCT116 were inoculated into 60-mm dishes (10^6^ cells/dish), and after 48 h of cell culture, 5 µL of Annexin V-FITC and 5 µL of PI were added to each dish and incubated for 30 min under light-protected conditions.

### Glucose Uptake, Lactate Production Levels Assay

The glycolytic capacity of the cells was assayed using a glucose uptake assay kit (#MAK083; Sigma-Aldrich, St. Louis, MO, USA) and lactate kit (#MAK064; Sigma-Aldrich). Cells were inoculated in 60-mm dishes (10^6^ cells/dish), and glucose consumption and lactate production in the cells were assayed after 48 h stable cell culture. Finally, all data were statistically analyzed.

### Western Blot Assay

Total proteins were extracted from CRC tissues, normal tissues and all cell lines using RIPA Lysis Buffer (#P0013C; Beyotime, Shanghai, China). The denatured proteins were then separated using SDS-PAGE (#P0670; Beyotime), and after electrophoresis, the protein bands were transferred to PVDF membranes (#FFP33; Beyotime). The PVDF membranes were placed in skimmed milk for containment treatment. Excess skim milk was further washed off the PVDF membranes using TBST buffer and co-incubated with primary antibodies. All primary antibodies were: proliferating cell nuclear antigen (anti-PCNA, ab29, Abcam, Cambridge, MA, USA), matrix metallopeptidase 2 (anti-MMP2, ab92536, Abcam, 1/2000), BCL2 associated X, apoptosis regulator (anti-Bax, ab32503, Abcam, 1/5000), hexokinase 2 (anti-HK2, ab209847, Abcam, 1/1000), beta-actin gene (anti-β-actin, ab8226, Abcam, 1/5000) and isochorismatase domain containing 1 (anti-ISOC1, orb400332, Biorbyt, Wuhan, China, 1/1000). The PVDF membranes were incubated overnight at 4 °C and washed again using TBST buffer at the end of the incubation. The PVDF membranes were then incubated with matched secondary antibodies (ab205718 and ab205719, Abcam, 1/20000). Finally, the PVDF membrane was color developed using ECL (#32132; Unique, Beijing, China), then photographed and stored.

### Dual-Luciferase Reporter Assay

The wild-type (WT) sequence segment of circ_0001821 and ISOC1 containing the putative miR-600 binding site, as well as the mutant-type (MUT) sequence segment of circ_0001821 and ISOC1 containing the mutated binding site of miR-600 were respectively synthesized and cloned into pmirGLO vector (Promega, Madison, WI, USA) to form circ_0001821^WT^, circ_0001821^MUT^, ISOC1-3'UTR^WT^ and ISOC1-3'UTR^MUT^ reporter vectors. The above plasmids were then co-transfected with miR-NC or miR-600 into SW620 and HCT116 cells. The assay of luciferase activity was performed after 48 h of co-culture. Finally, statistical analysis was performed.

### RNA Pull-Down Assay

The interaction between circ_0001821 and miR-600 was analyzed using RNA pull-down kit (#KT103-01; Gzscbio, Guangzhou, China). Biotin-labeled miR-600 (Bio-miR-600) and miR-NC (Bio-miR-NC) provided by Ribobio were first transfected into SW620 and HCT116 cells. Then they were co-incubated with streptavidin magnetic beads. After incubation, total RNA was harvested by eluting the RNA-binding complex from the magnetic beads. Then the RNA was extracted and the enrichment of circ_0001821 was detected and normalized.

### Animal Experimentation

In this study, all mice (Balb/c, 6-week-old) were purchased from Vital River (Beijing, China). Short hairpin RNA targeting circ_0001821 (sh-circ_0001715) and control (sh-NC) were synthesized and inserted into lentiviral vector by Geneseed, followed by lentiviral packaging. SW620 cells after lentiviral infection were inoculated by subcutaneous injection into nude mice. The volume of the tumors (length × width ^2^ × 1/2) was measured at 7-day intervals, and then on day 35, after the volume measurements were completed, the mice were executed and the mass of the tumors was weighed. All animal experiments were approved by the Shannxi Provincial People's Hospital Research Animal Care and Use Committee.

### Immunohistochemistry (IHC)

The expression of Ki-67 in mouse tumor tissues was assessed using IHC. Tumor tissues were removed and then washed with PBS, followed by sectioning. The tumor tissues were then incubated with primary antibody Ki-67 (anti-Ki-67, 1:200, Abcam) overnight at 4 °C. Then the secondary antibody was added and incubated for 30 min at 37 °C. Finally, the sections were stained with hematoxylin (#H9627; Sigma-Aldrich) and sealed. Finally, image acquisition was performed using a microscope.

### Statistical Analysis

All experiments were performed with three replications. All data were counted, analyzed and then plotted using GraphPad Prism 8 (GraphPad, La Jolla, CA, USA). Two independent sets of data were analyzed using the Student's *T*-test. More than two groups were analyzed using one-way ANOVA or two-way ANOVA. The correlation analysis among circ_0001821, miR-600 and ISOC1 was performed using Pearson’s correlation analysis. *P* values were considered statistically significant when they were less than 0.05.

## Results

### Circ_0001821 was UpRegulated in CRC Tissues

**Fig. 1:**
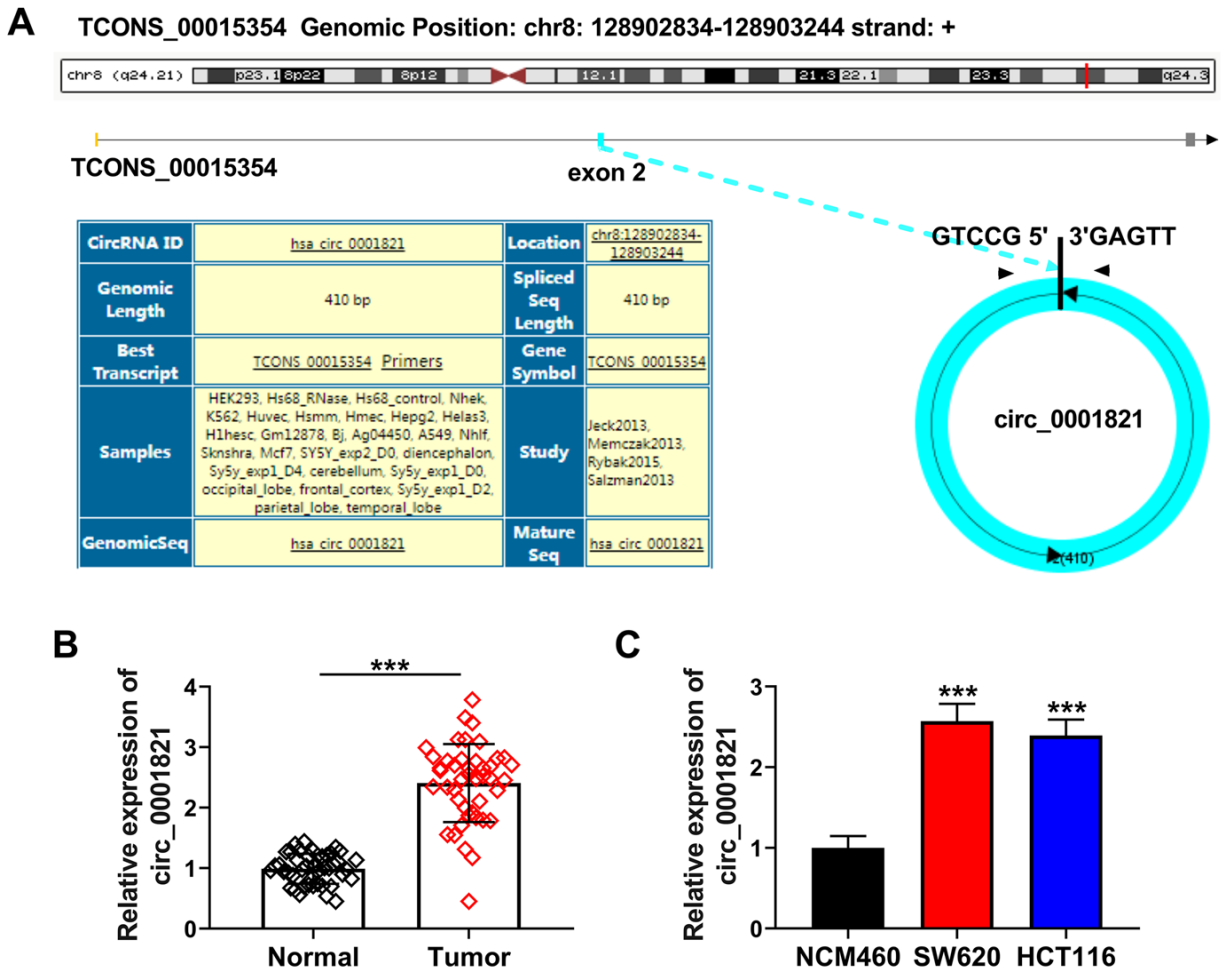
Circ_0001821 was increased in CRC tissues. **A** The chromosomal location of circ_0001821 on its host gene TCONS_00015354 was shown, along with the exons contained in circ_0001821. **B** and **C** The expression of circ_0001821 was tested in CRC tissues, CRC cell lines (SW620 and HCT116), and matched normal controls by RT-qPCR. ***p < 0.001

To investigate the role played by circ_0001821 in the process of CRC, we analyzed the structure of circ_0001821. The analysis showed that circ_0001821 was chromosome 8 located on its host gene TCONS_00015354 (chr8: 128902834-128903244) and was composed of 1 exon (Fig. 1A). Further, the expression of circ_0001821 was examined in the tissues of 44 CRC patients. The results showed that the expression of circ_0001821 was significantly upregulated in CRC tissues compared to normal tissues (Fig. 1B). Finally, we examined the expression in CRC cell lines, and the results showed that the expression of circ_0001821 was also significantly increased in SW620 and HCT116 cells compared with normal NCM460 cells (Fig. 1C). In summary, the expression of circ_0001821 was elevated in both CRC tissues and its cell lines.

### Silencing circ_0001821 Inhibited Cell Proliferation, Migration, Invasion and Glycolytic Capacity as Well as Induced Apoptosis in CRC Cell Lines

**Fig. 2:**
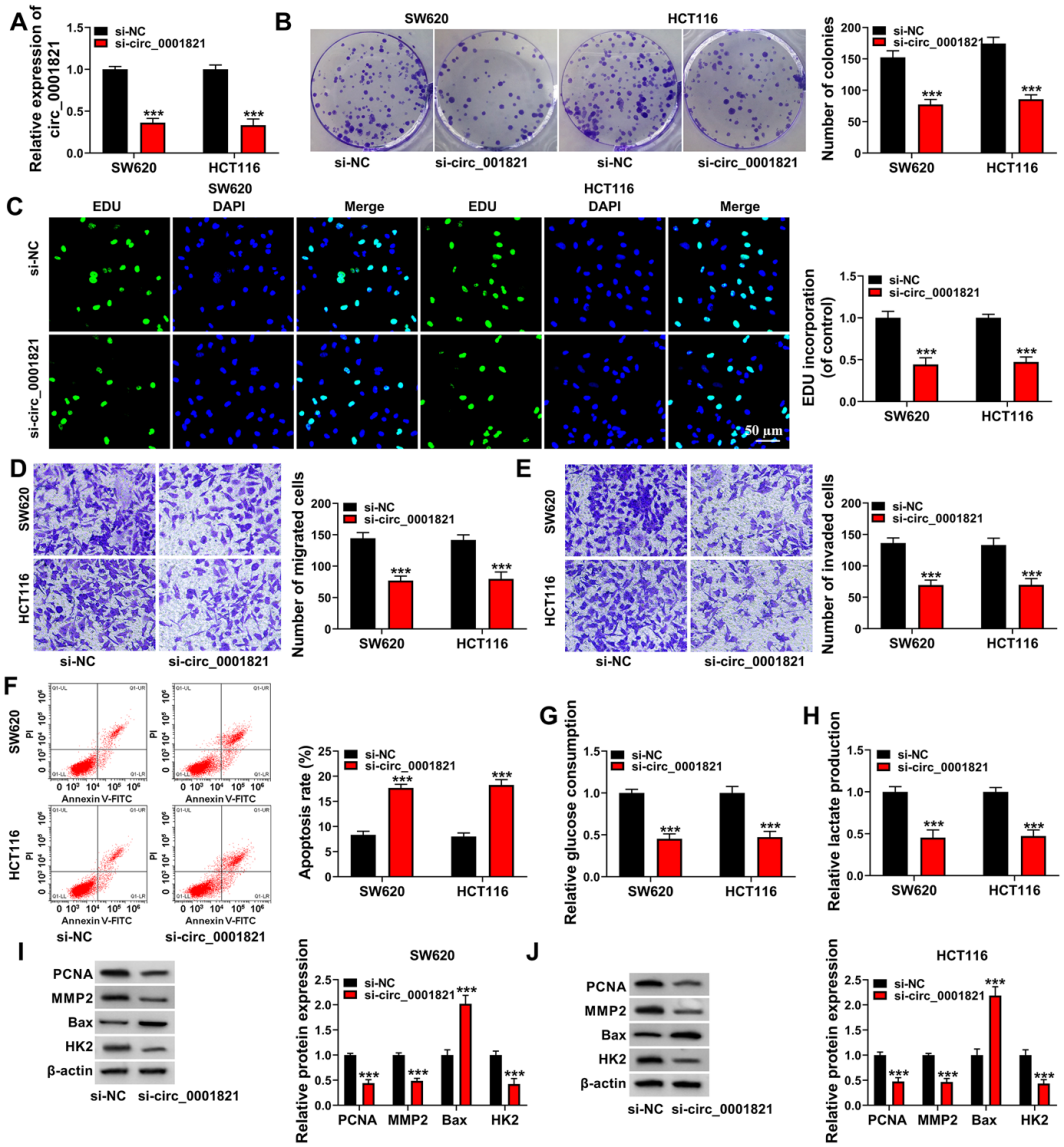
Effect of circ_0001821 silencing on the biological properties of CRC cell lines. **A** The knockdown efficiency of circ_0001821 was detected by RT-qPCR in SW620 and HCT116 cells. **B** and **C** The effect of silencing circ_0001821 on cell proliferation was assessed by colony formation assay and EDU assay. **D** and **E** The effect of silencing circ_0001821 on the ability of cells to migrate and invade was examined by transwell assay. **F** The effect of knockdown of circ_0001821 on the rate of apoptosis was measured by flow cytometry. **G** and **H** Glucose uptake and lactate production in CRC cells were detected after silencing circ_0001821. **I** and **J** The protein expression of PCNA, MMP2, Bax and HK2 was detected by western blot assay. ***p < 0.001

We further investigated the biomolecular role played by circ_0001821 in CRC cell lines. We first examined the knockdown efficiency of circ_0001821 in CRC cell lines, the RT-qPCR showed that the expression of circ_0001821 was significantly reduced in CRC cells after si-circ_001821 transfection (Fig. 2A). Immediately after silencing circ_0001821, the proliferation ability of the SW620 and HCT116 cells was significantly decreased (Fig. 2B, C). Further, we assessed the migration and invasion ability of the cells, transwell assay results showed that the migration ability and invasion abilities of the cells were significantly inhibited after silencing circ_0001821 (Fig. 2D, E). Also, we examined that the apoptosis of cells was significantly increased after silencing circ_0001821 (Fig. 2F). In addition, the glucose consumption and lactate production of the cells were also decreased after circ_0001821 silencing (Fig. 2G, H). Last but not least, to confirm the results of the above assay, we also examined the marker proteins of proliferation (PCNA), metastasis (MMP2), apoptosis (Bax) and glycolysis (HK2) and obtained consistent results (Fig. 2I, J). Overall, silencing circ_0001821 in CRC cell lines inhibited proliferation, migration, invasion and glycolysis, while apoptosis was promoted.

### Circ_0001821 Acted as a Sponge for miR-600

**Fig. 3:**
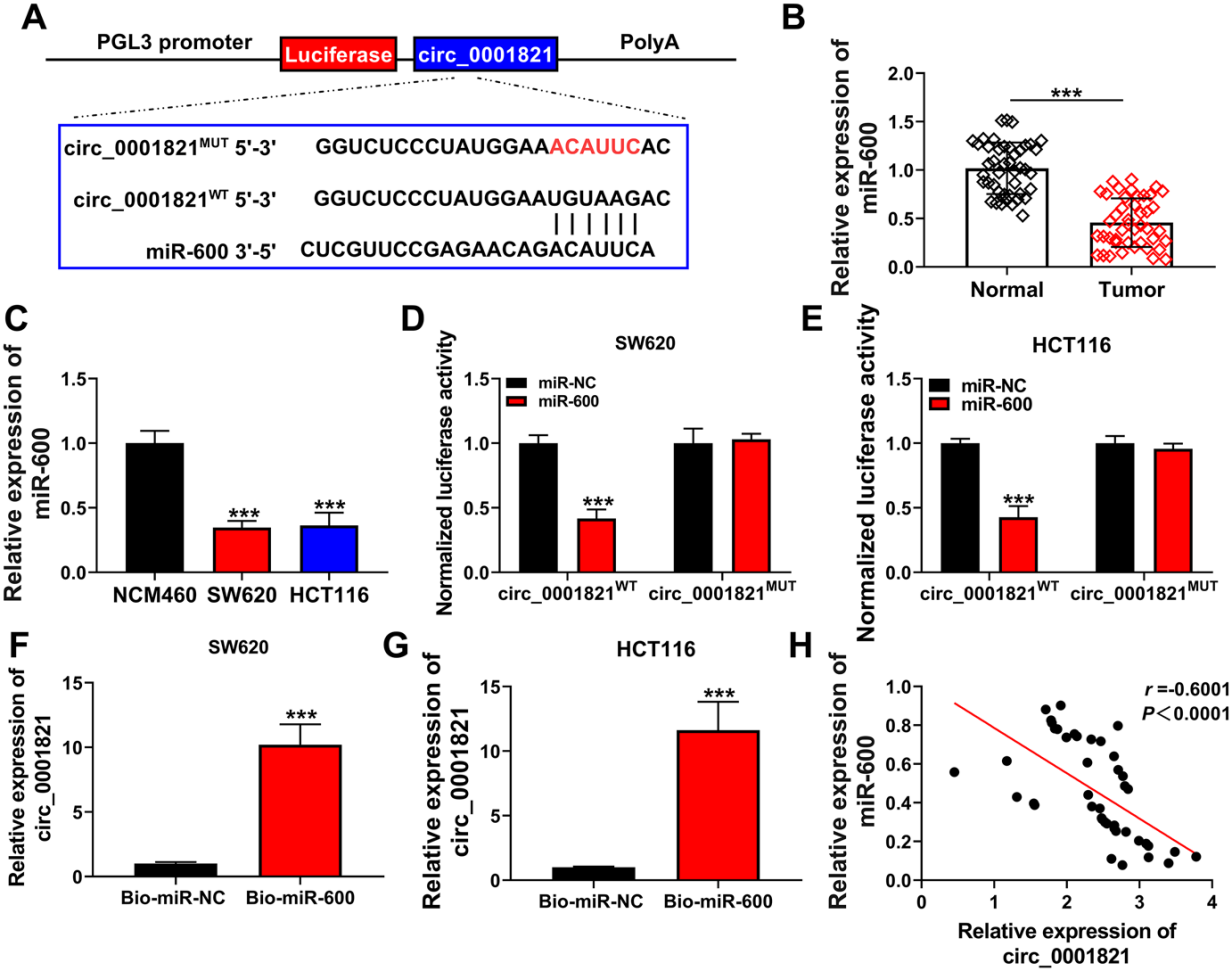
Identification of miR-600 as a potential miRNA sponge for circ_0001821. **A** Binding sites and mutant sites between circ_0001821 and miR-600 were shown. **B** The expression of miR-600 in tumor and normal tissues was assessed by RT-qPCR. **C** The expression of miR-600 in NCM460, SW620 and HCT116 cells was assessed by RT-qPCR. **D**–**G** Interaction between circ_0001821 and miR-600 was detected by dual-luciferase reporter assay and RNA pull-down assay. **H** Pearson’s correlation analysis showed the correlated expression levels between circ_0001821 and miR-600. ***p < 0.001

To further investigate the molecular mechanism of circ_0001821 in CRC development, we predicted the downstream targeting regulators of circ_0001821 using circinteractome website (https://circinteractome.nia.nih.gov/api/v2/mirnasearch?circular_rna_query=hsa_circ_0001821&mirna_query=&submit=miRNA+Target+Search). The results showed that miR-600 might be a downstream target of circ_0001821. Circ_0001821 and miR-600 had corresponding binding sites, and to further investigate the relationship between them, we mutated the binding sites of circ_0001821 and miR-600 (Fig. 3A). We examined the expression of miR-600 in CRC tissues and cell lines, and miR-600 expression was significantly reduced (Fig. 3B, C). Dual-luciferase reporter assay displayed a decrease of luciferase activity in SW620 and HCT116 cells with circ_0001821^WT^ and miR-600 mimic cotransfection (Fig. 3D, E). RNA pull-down assay displayed that high abundance of circ_0001821 was enriched by Bio-miR-600 probe relative to Bio-miR-NC probe (Fig. 3F, G). Finally, the correlation analysis showed a significant negative correlation between circ_0001821 and miR-600 (Fig. 3H). Overall, the expression of miR-600 was reduced in CRC tissues as well as CRC cell lines, and in addition there was an interaction between circ_0001821 and miR-600.

### The Regulatory Effect of Silencing circ_0001821 on Cells Could be Counteracted by miR-600 Inhibitor in CRC Cell Lines

**Fig. 4:**
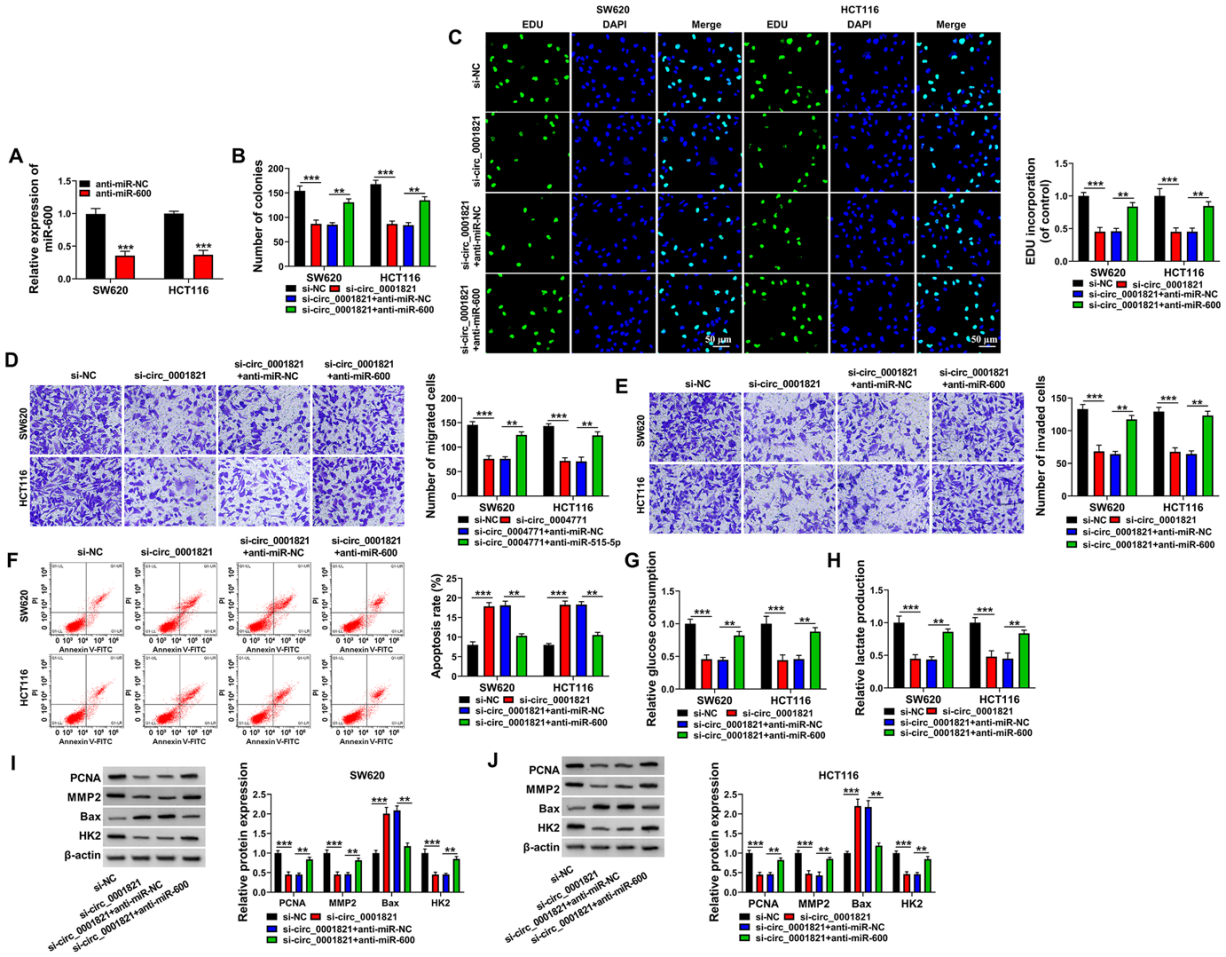
Effect of circ_0001821 silencing on cell biological properties was reversed by miR-600 inhibitor. **A** The expression of miR-600 was tested by RT-qPCR in CRC cell lines. **B** and **C** The proliferative capacity of the cells was assayed by colony formation assay and EDU assay after transfection with si-NC, si-circ_0001821, si-circ_0001821 + anti-miR-NC or si-circ_0001821 + anti-miR-600 in SW620 and HCT116 cells. **D** and **E** Cell migration and invasion capabilities in abovementioned cells were measured by transwell assay. **F** The apoptotic capacity of the abovementioned cells was detected by flow cytometry. **G** and **H** Glucose consumption and lactate production in SW620 and HCT116 cells were assessed. **I** and **J** The protein expression of PCNA, MMP2, Bax and HK2 was detected in abovementioned cells using western blot. **p < 0.01, ***p < 0.001

Firstly, we examined the knockdown efficiency of miR-600 in SW620 and HCT116 cells, and the results showed that miR-600 expression was reduced (Fig. 4A). Colony formation assay and EDU assay on cell proliferation ability showed that silencing circ_0001821 significantly inhibited cell proliferation, but was counteracted by the inhibitor of miR-600 (Fig. 4B, C). In addition, the effect of silencing circ_0001821 on cell migration and invasion abilities as well as apoptosis was reversed by miR-600 inhibitor (Fig. 4D–F). Further we explored the glycolytic capacity of SW620 and HCT116 cells, the results showed that silencing circ_0001821 inhibited cellular glucose consumption and lactate production, but these were counteracted by low expression of miR-600 (Fig. 4G, H). Finally, we quantified the marker proteins for the above functions and showed that the inhibitory effects of PCNA, MMP9 and HK2 inhibited by silencing circ_0001821 and the promotive effects of Bax were reversed by the low expression of miR-600 (Fig. 4I, J). In summary, the effects of low expression of circ_0001821 on the biological properties of cells could be reversed by miR-600 inhibitor.

### ISOC1 was the Target Gene of miR-600

**Fig. 5:**
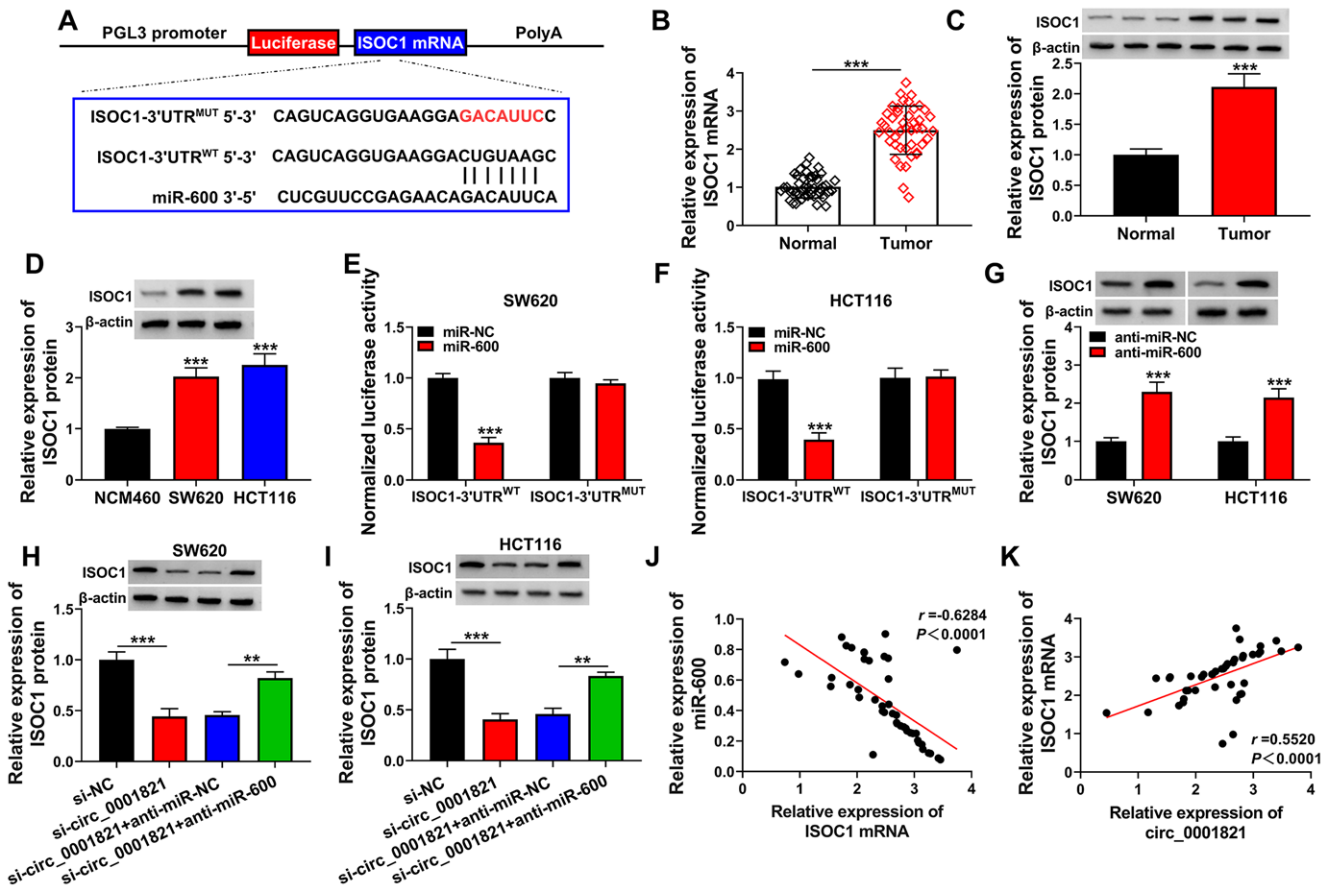
MiR-600 directly bound to ISOC1. **A** The binding site between miR-600 and ISOC1 mRNA was shown. **B**–**D** The mRNA and protein expression of ISOC1 was detected by RT-qPCR and western blot assay. **E** and **F** Interaction relationship between miR-600 and mRNA was measured by dual-luciferase reporter assay. **G** The protein expression of ISOC1 was measured after transfection with miR-NC or anti-miR-600. **H** and **I** The protein expression of ISOC1 was measured by western blot assay after transfection with si-NC, si-circ_0001821, si-circ_0001821 + anti-miR-NC or si-circ_0001821 + anti-miR-600. **J** and **K** Pearson’s correlation analysis showed the correlated expression levels between miR-600 and ISOC1 and between circ_0001821 and ISOC1 were shown. **p < 0.01, ***p < 0.001

We explored the regulatory network of circ_0001821/miR-600 in cells in more depth. Bioinformatics software (TargetScan: https://www.targetscan.org/cgi-bin/targetscan/vert_72/targetscan.cgi?species=Human&gid=&mir_sc=&mir_c=&mir_nc=&mir_vnc=&mirg=miR-600) was used to detect the downstream target genes of miR-600. The results showed that ISOC1 might be a downstream regulator of miR-600. The binding sites of miR-600 and ISOC1 are shown in Fig. 5A. In addition, the expression of ISOC1 was significantly increased in CRC tissues as well as in its cell lines (Fig. 5B–D). The results of dual-luciferase reporter assay showed that miR-600 and ISOC1 interacted with each other (Fig. 5E, F). The protein expression of ISOC1 was significantly increased after inhibition of miR-600 (Fig. 5G). In addition, the protein expression of ISOC1 was decreased with the decrease of circ_0001821, but the protein expression of ISOC1 increased significantly after the addition of miR-600 inhibitor (Fig. 5H, I). Correlation analysis showed that miR-600 and ISOC1 expression were negatively correlated, and circ_0001821 and ISOC1 expression were significantly positively correlated (Fig. 5J, K). On all accounts, miR-600 could regulate the expression of ISOC1, and in addition, circ_0001821 could regulate the expression of ISOC1 through miR-600.

### The Regulatory Effect of circ_0001821 Knockdown on Cell Biological Properties Could be Reversed by Overexpression of ISOC1

**Fig. 6:**
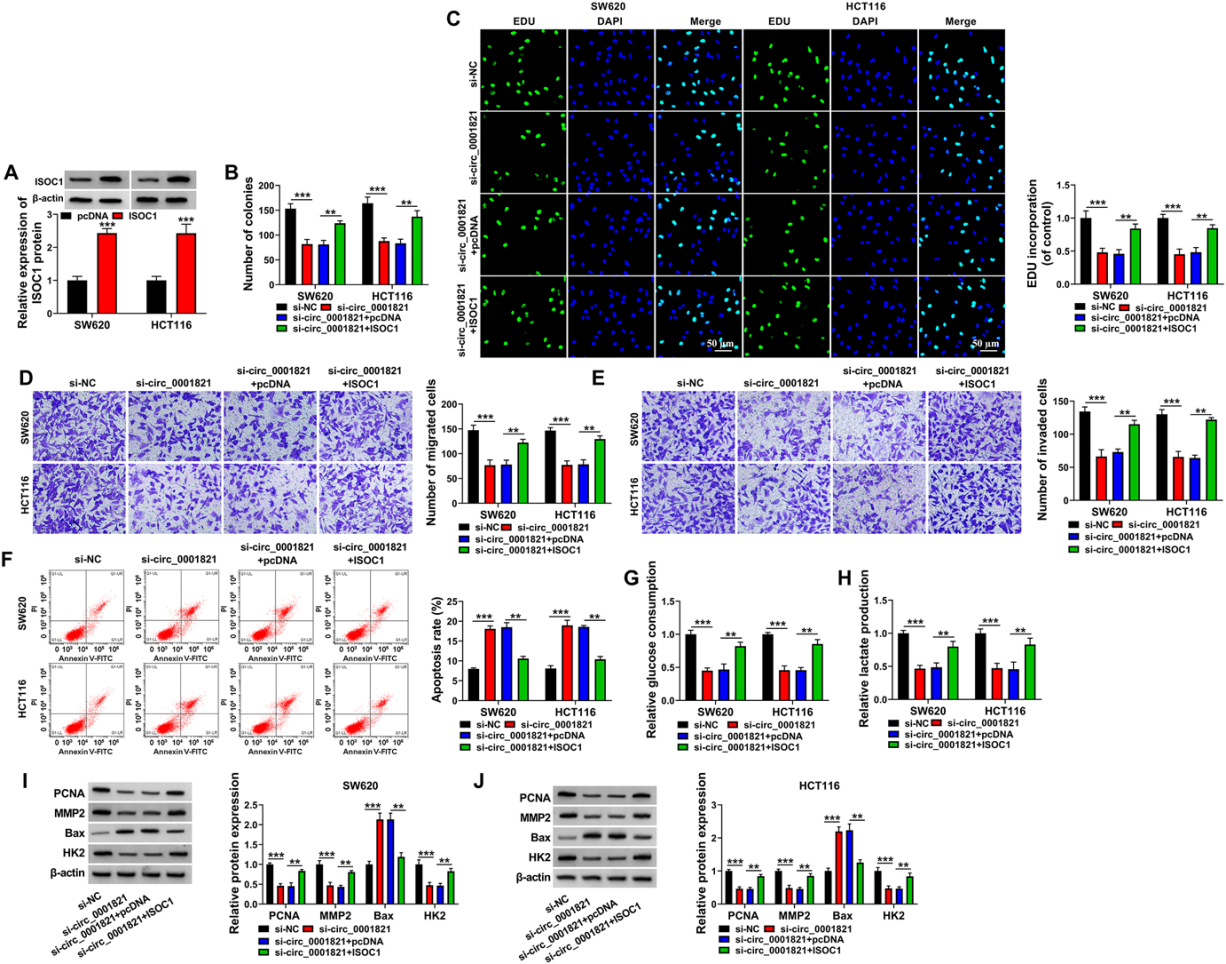
Effect of circ_0001821 silencing on cell biological properties was counteracted by overexpression of ISOC1. **A** Overexpression efficiency of ISOC1 was examined by western blot assay. **B**–**J** SW620 and HCT116 cells were transfected with si-NC, si-circ_0001821, si-circ_0001821 + pcDNA or si-circ_0001821 + ISOC1. **B** and **C** The proliferative capacity of the cells was measured by colony formation assay and EDU assay. **D** and **E** The cell migration and invasion capabilities were assessed by transwell assay. **F** Flow cytometry was used to detect apoptosis of cells. **G** and **H** Glucose consumption and lactate production in CRC cell lines were tested. **I** and **J** Western blot assay was used to assess the protein expression of PCNA, MMP2, Bax and HK2. **p < 0.01, ***p < 0.001

We investigated the regulatory network of circ_0001821 in CRC development in more depth. First, we overexpressed ISOC1 in SW620 and HCT116 cells, and the results showed that the expression of ISOC1 was significantly increased (Fig. 6A). Silencing circ_0001821 inhibited the proliferative capacity of cells, which was reversed by overexpression of ISOC1 (Fig. 6B, C). In addition, the inhibitory effects of low expression of circ_0001821 on both the migration and invasive abilities of cells were counteracted by high expression of ISOC1 (Fig. 6D, E). The apoptotic rate of cells was increased with the decrease of circ_0001821, but this effect was inhibited again after overexpression of ISOC1 in the cells (Fig. 6F). In addition, silencing circ_0001821 inhibited the glycolytic capacity of cells, but this was counteracted by increased expression of ISOC1 (Fig. 6G, H). Finally, we examined the above-mentioned signature proteins and obtained the same results (Fig. 6I, J). To sum up, the effects of silencing circ_0001821 on cell biological functions could be reversed by high expression of ISOC1.

### Circ_0001821 Silencing Hampered Tumor Growth In Vivo

**Fig. 7:**
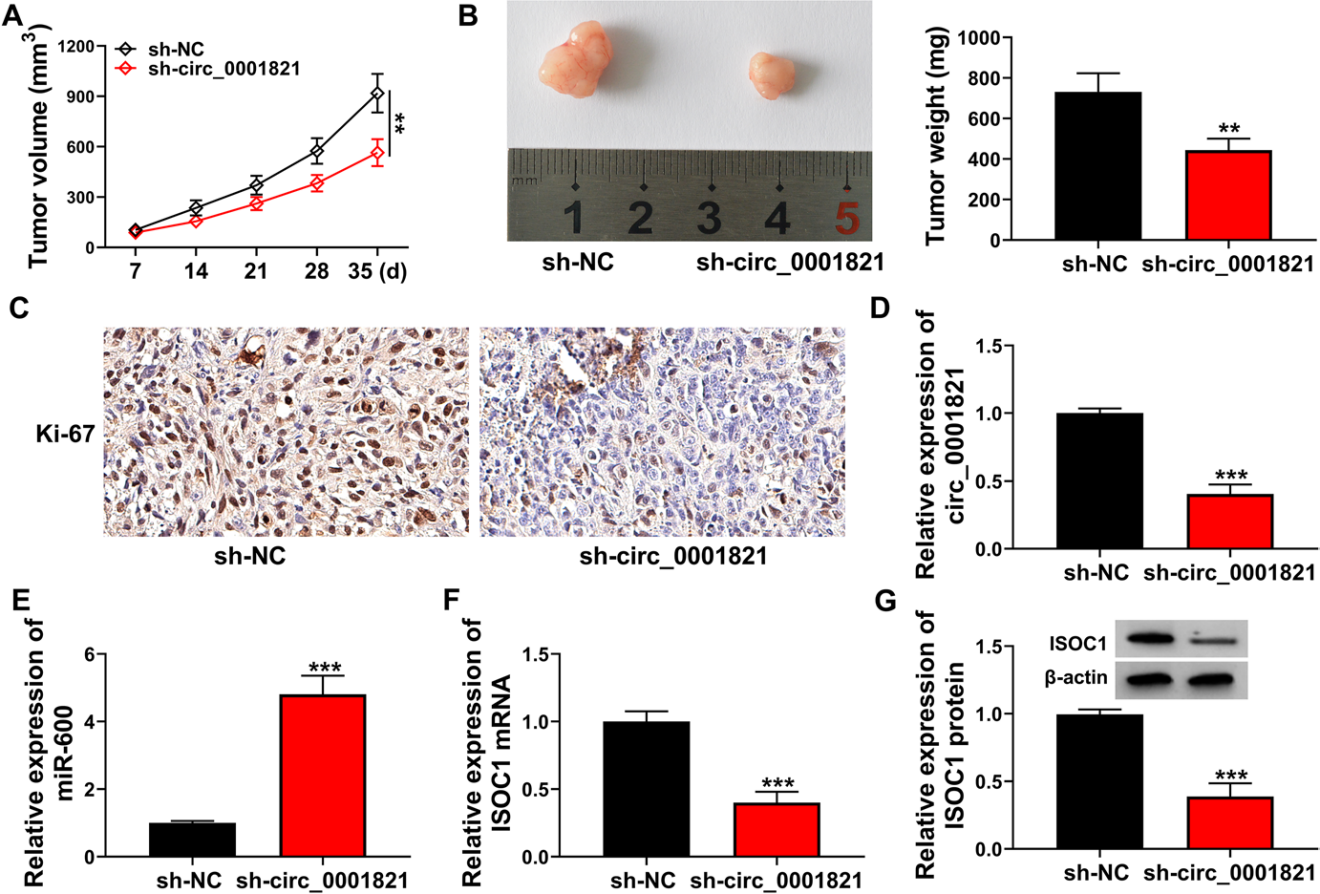
Knockdown of circ_0001821 repressed tumor growth in vivo. **A** and **B** Tumor volume and tumor weight in sh-NC group and sh-circ_0001821 group were measured. **C** Ki-67 levels were assessed by IHC. **D** and **E** The expression of circ_0001821 and miR-600 was assessed by RT-qPCR. **F** and **G** The mRNA and protein expression of ISOC1 was tested by RT-qPCR and western blot assay. **p < 0.01, ***p < 0.001

To verify the role of circ_0001821 in vitro, we investigated the function of circ_0001821 in vivo. The results of the mouse xenograft tumor model assay showed that the tumor volume and weight were significantly suppressed in the sh-circ_0001821 group compared to the control group (Fig. 7A, B). The results of IHC analysis showed that the expression of Ki-67 was significantly reduced in the sh-circ_0001821 group compared with the control group (Fig. 7C). Finally, we examined the expression of circ_0001821, miR-600 and ISOC1 in sh-circ_0001821, and the results showed that the expression of circ_0001821 and ISOC1 was significantly reduced, while the expression of miR-600 was significantly increased (Fig. 7D–G). The above results suggested that circ_0001821 inhibited tumor growth in vivo. It further indicates that circ_0001821 plays an active role in the growth of tumors in vivo.

## Discussion

Earlier or more sensitive biomarker to diagnose CRC is very good for CRC patients (Yang et al. 2017). A growing number of studies have shown that circRNAs were significantly dysregulated and had a very important regulatory role in many diseases, such as hepatocellular carcinoma (Chen et al. 2021c), cardiovascular disease (Zhang et al. 2021), non-small-cell lung cancer (Chen et al. 2021b), prostate cancer (Mugoni et al. 2021) and breast cancer (Wu et al. 2021). In our study, we determined the expression of circ_0001821 in CRC tissues and its cell lines, and investigated more deeply the biological function of circ_0001821 played in CRC development. It provided a valid biomarker for better confirming the diagnosis of CRC.

Another important aspect was that circRNAs could regulate cancer progression and played a regulatory role in cancer progression. For instance, the expression of circ_002178 was increased in CRC, and silencing circ_002178 inhibited the proliferative capacity and glycolytic capacity of cells (Xu et al. 2021); circIPO11 was significantly increased in hepatocellular carcinoma, and in addition, silencing circIPO11 inhibited the ability of cancer cells to migrate and metastasize (Gu et al. 2021). A significant increase in circ_0001821 expression in CRC tissues has been reported (Song et al. 2021). Consistent results were obtained in our study, circ_0001821 expression was significantly increased in both CRC tissues and cell lines. We further investigated the function of circ_0001821 in CRC cell lines. We silenced circ_0001821 in CRC cell lines and the results showed that knockdown of circ_0001821 significantly inhibited the proliferation, migration, invasion and glycolytic capacity of cancer cells as well as induced apoptosis. Our findings further suggested that circ_0001821 promoted the developmental process of CRC.

CircRNAs that have binding sites for miRNAs may act as sponges for miRNAs to play regulatory roles (Qu et al. 2021). In addition, more and more studies had demonstrated that miRNAs regulated the expression of downstream target genes (Bi et al. 2019; Zhang et al. 2021), and circRNAs further regulated the expression changes of downstream genes through miRNAs (Bi et al. 2019). In this study, our experimental results showed that circ_0001821 could bind and interact with miR-600. Our results in functional assays showed that the effects of circ_0001821 silencing on the biological functions of cells could be reversed by miR-600 inhibitor. The above results suggested that circ_0001821 regulated the pathogenic process of CRC through miR-600. Of note, previous studies reported that RNA-binding protein could also act as “miRNA sponge” to sequester miRNAs and thus mediate the translation of downstream functional genes (Poria et al. 2016). For instance, HuR RNA-binding protein sequestered miR-21 to regulate the translation of PDCD4 gene (Poria et al. 2016). RNA-binding proteins that may govern miR-600 expression should be investigated to further understand the role of miR-600 in CRC.

ISOC1 is a hydrolase and belongs to protein-coding genes (Gao et al. 2020). Many studies have shown that ISOC1 was positively correlated in tumor progression. ISOC1 has been reported to be highly expressed in lung cancer (Shi et al. 2021), gastric cancer (Zhao et al. 2020), colon cancer (Gao et al. 2020) and pancreatic cancer (Cheng et al. 2019) and also promotes the cancer process and growth of cancer cells. These findings suggested that ISOC1 might be an oncogene. Therefore, we concluded that ISOC1 was a downstream target gene of miR-600 by bioinformatics analysis. Furthermore, circ_0001821 could regulate the expression of ISOC1 through miR-600. It was further concluded by rescue assay that the effect of circ_0001821 silencing on CRC cell lines could be counteracted by overexpression of ISOC1.

To sum up, in this study, we demonstrated the upregulation of circ_0001821 expression in CRC samples. Silencing circ_0001821 suppressed cell proliferation, migration, invasion and glycolytic capacity, while promoted apoptosis in CRC cell lines. Circ_0001821 could further regulate ISOC1 expression by sponging miR-600. Mechanistically, the effects of circ_0001821 silencing on cell biological properties could be reversed by miR-600 inhibitor or overexpression of ISOC1. These results suggested that circ_0001821 could be used as a molecular marker for CRC and that this study might provide a useful theoretical basis for earlier and more effective confirmation of CRC diagnosis.

## Data Availability

The analyzed data sets generated during the present study are available from the corresponding author on reasonable request.
